# Physical Self-Concept and Physical Activity Levels in University Students during the COVID-19 Pandemic: A Cluster Analysis

**DOI:** 10.3390/ijerph19052850

**Published:** 2022-03-01

**Authors:** Daniel Duclos-Bastías, Frano Giakoni-Ramírez, Daniel Martínez-Cevallos

**Affiliations:** 1 Escuela de Educación Física, Pontificia Universidad Católica de Valparaíso, Valparaiso 2374631, Chile; 2 Faculty of Education and Social Sciences, Universidad Andres Bello, Santiago 7550000, Chile; frano.giakoni@unab.cl or; 3 Facultad de Ciencias Económicas y Administrativas, Universidad Católica de la Santísima Concepción, Concepcion 4070129, Chile; martinezcevallos1988@gmail.com

**Keywords:** physical self-concept, clusters, COVID-19, university students, physical activity

## Abstract

With the COVID-19 pandemic, the physical activity (PA) levels of university students declined as a result of confinement. The aim of the study was to analyse the segmentation of university students according to physical self-concept ratings and to determine the differences between each cluster during the pandemic. The sample consisted of 492 students aged 18–31 years, 36.8% male and 63.2% female, who were administered the PSDQ-S and IPAQ instruments. The data collected were analysed with SPSS software, from which descriptive statistics, a cluster analysis from the PSDQ-S, was obtained. The IPAQ and socio-demographic variables were used to characterise the groups. Finally, a non-hierarchical K-means analysis was performed to establish the clusters. The results reported three different group profiles of students. Significant differences were found in all self-concept variables analysed, with the exception of some items. In relation to PA level, it could be established that the Positive Physical Self-Concept group had the highest PA level and was composed of 52.1% females and 34.4% males, showing a high physical self-concept, whereas, in the Medium-Physical Self-Concept and Negative-Physical Self-Concept groups, females were predominant in number. They were also the least physically active groups and had a low physical self-concept.

## 1. Introduction

The global context during the year 2020 has been characterized by different social and health changes as a consequence of the pandemic caused by the SARS-CoV-2 virus, an emergency declared by the World Health Organization on 11 March 2020 [1]. One of the main measures implemented to reduce the spread of the virus in Chile and other countries of the world has been social isolation through protective measures such as quarantine, confinement, reduction in mobility and reduction in capacity to avoid crowds. These measures have led to the closure of gyms and sports centres, and the prohibition of sports practice. This has had an impact on the regularity of physical activity of the population, in the same line as that indicated by Mera et al. [2], who state that the decrease in energy expenditure is affected by staying at home and sedentary behaviours such as sitting or lying down for an extended period of time and attending to academic or work responsibilities.

In Chile, according to the results of the National Survey of physical activity and sport habits in the population aged 18 years and over, the following figures were obtained [3], 81.3% of the Chilean population aged 18 years or older is physically inactive. This is a negative outlook, since the practice of physical activity brings with it multiple benefits of a social, physical, cognitive, and motor nature, among others [4]. There is abundant evidence on the decrease in physical-sports activity; this fact generates a great social concern due to its negative effects on people’s well-being, specifically for the physical and mental health associated with such activity [5,6]. Physical activity not only seems to be related to better physical health, as is commonly believed, but has also been shown to influence an active lifestyle on people’s psychological and emotional well-being [7].

Among the psychological benefits of physical activity, which are independent of age or physical condition, exercise helps us to improve our physical self-concept [7,8,9].

Physical self-concept is highly related to physical activity, with the result that students who practice physical activity on a regular basis have a better conception of their physique than those who do not. Self-concept is defined by how one sees oneself and self-esteem by how one values and appreciates what one sees [10]. This is of great importance, for example, in the development of personality, which plays a very important role in the future employment of university students, since the higher the level of self-concept, the higher the level of social development, the better the professional and personal performance [11]. In relation to the group under study in the present research, university students are defined according to Coba as follows [12], as “people who have managed to enter an excluding social model that enrolls them in an academic process, oriented towards the performance of a profession”, so that the environment in which they develop is a determining factor in a series of habits and attitudes that will have an impact on their future.

While preventive measures that have been adopted in most countries of the world are fundamental in the fight against COVID-19, such as isolation including confinement or social distancing, while contributing to the distribution and care of cases that can be covered by the health system, it has also been argued that it may encourage sedentary behaviour, reduce regular physical activity or avoid daily activities, which increases the risk of developing diseases or worsening previous pathologies [13]. Therefore, several studies have been conducted to determine the real impact of confinement measures on physical activity in the university population [14,15,16].

Given that the central aim of this project is to understand the effects of the pandemic on the physical self-concept and its relationship with physical activity in young Chilean university students, it is necessary to establish a baseline and conceptual axes on which to base our understanding of it.

Along the lines established by Cervantes and Gaete [17], during the transition from childhood to adolescence, profound physical and emotional changes take place, which are often difficult and accentuate the negative emotions of those who experience them. These changes are later reflected in adulthood, where they forge their identity during the adolescent stage, an identity that is still being formed, so the changes are still present but not in such a conflictive way as before. From a social dimension, according to Garrido and Requena [18], youth is a period of transition and passage to adulthood. One of the characteristics of this period is emancipation, which materialises in a set of attitudes of integration in which responsibilities are left behind, and the acceptance and development of new roles associated with new responsibilities assigned to the adult begins, something Arnett [19] refers to the concept of emerging adulthood. Thus, one could speak of the social destiny of being an adult as integration. According to this point of view, the population is in a phase of life in which forms of bonding and participation within the social organisation and the development of independent thinking are established [20], which makes young people become incorporated as social persons. In the presence of this process, social destructuring is more than evident and, therefore, forms of social exclusion rather than inclusion would be manifested and, with it, people who could be ceasing to participate as citizens in the context of adulthood [21].

In the literature it is pointed out that, precisely at the university stage, there are many changes in the lifestyle of students, many have to move to other cities where the university institutions are located, there is also an increase in the levels of demand and dedication to their studies, less rest and more responsibilities, but also more freedom to spend their free time in different ways together with their significant others [22,23,24]. Pavón and Moreno [25] and Dosil [26], these factors, together with the decrease in motivation levels towards physical activity that occurs at this stage, can lead to the abandonment of sport, either temporarily or permanently.

On the other hand, within the framework of the present study and in relation to all the changes involved, different authors agree that self-concept is made up of multiple dimensions, encompassing different areas that together form the self-concept. For Pastor, Balaguer, and García−Merita [27], self-concept is one of the factors that influence the configuration of the personality, a construct linked to various elements and experiences of each person for their development; in the same way, the perception of self-concept is linked to psychological well-being and intervenes in the performance of various actions, sometimes causing serious repercussions for the healthy state of the person, particularly in relation to physical self-concept, as its positive influence has been shown on healthy behaviours such as physical activity [28,29,30]. In the same line, Marchago [31] includes perceptual and cognitive elements such as physical features, body size and shapes, and other representations of body-related aspects such as appearance, health, and weight. While Esnaola and Revuelta [32] even incorporate one’s own perception of physical ability, strength, and fitness.

Therefore, self-concept can be understood as a complex construct that encompasses many other areas such as self-esteem and body image, so physical activity contributes to increased self-esteem and self-confidence, decreased levels of anxiety, anger, distress, and depression, among others, reduced fatigue and a perceived sense of well-being [33].

Along the same lines as those pointed out by Sánchez [34], reinforcement of self-esteem as a result of physical exercise, with physical activity having an impact on these psychological aspects mentioned above, occurs in a similar way regardless of age [35,36,37].

In order to achieve the purpose of the present study (analyse the segmentation of university students according to physical self-concept ratings and to determine the differences between each cluster during the COVID-19 pandemic), two hypotheses have been put forward that can be tested after cluster analysis:

**Hypothesis** **1** **(H1).** *There are different clusters representing different profiles of university students in relation to physical self-concept*.

**Hypothesis** **2** **(H2).** *The different clusters found show significant differences in terms of physical self-concept variables such as: physical activity, appearance, body fat, coordination, endurance, flexibility, health, sport, strength, global physique, and global esteem*.

## 2. Materials and Methods

The present study is defined as a non-experimental descriptive-correlational study. A total of 492 university students participated with an age range between 18 and 31 years, distributed by sex: 181 men (36.8%) and 311 women (63.2%). A total of 81.9% resided in Valparaíso, with 17.5% residing in other regions and 0.6% outside Chile. Finally, with regard to the type of housing, 76.2% lived in a house and 23.8% in a flat. For the selection of the sample, a non-probabilistic convenience sample was chosen, considering the current context and access to the sample units.

### 2.1. Instruments

For the data collection, the Physical Self-Concept Assessment Test was applied through the adapted and validated Spanish version of the short form of the Physical Self-Description Questionnaire (PSDQ-S) by Marsh, Martin, and Jackson [38] and translated into Spanish [39]. The PSDQ-S was designed to measure global physical self-concept and global esteem, as well as 40 items and nine dimensions related to physical self-concept: physical activity, appearance, body fat, coordination, endurance, flexibility, health, sport, and strength, using a 5-level Likert scale, with 1 being “strongly disagree” and 5 being “strongly agree”.

To determine and classify the level of physical activity, the long version (31 items) of the self-administered format of the International Physical Activity Questionnaire (IPAQ) of Craig et al. [40] and translated into Spanish [41], was used with the following levels of daily activities:

1. High: report 7 days per week of any combination of walking, moderate, or high intensity activity achieving a minimum of 3000 MET-min/week; or when vigorous activity was reported on at least 3 days per week achieving at least 1500 MET-min/week.
2. Moderate: reporting 3 or more days of vigorous activity for at least 20 min per day; or when 5 or more days of moderate activity and/or walking for at least 30 min per day; or 5 or more days of any combination of walking and moderate or vigorous activity achieving at least 600 MET-min/week.
3. Low: was defined when the subject’s level of physical activity did not fall into the high or moderate categories.

### 2.2. Procedure

An email inviting voluntary participation was sent to students of the Pontificia Universidad Católica de Valparaíso enrolled in 2020 in undergraduate and postgraduate courses. The nature and scope of the study was explained in the invitation. The students who positively agreed to participate, accessed a link that directed them to complete an informed consent form adhering to the Singapore Declaration [42] and Helsinki Statement [43], which had to be signed in order to proceed with the instruments described above; 492 valid responses were received with signed informed consent and instrument via the online platform. The information was collected between August and September 2020.

It should be noted that the gradual strategy “Step by Step we take care of ourselves” was presented at the end of July 2020, with the purpose of creating a common framework for the systematization of the planning of restrictions and sanitary norms, according to the epidemiological situation at the territorial level. During data collection the Valparaíso region was in a step 2 restriction, in which activities in gyms and similar must always be 2 m between machines, and all attendees must have their Mobility Pass. For activities in open spaces there is a maximum of 10 people.

### 2.3. Statistical Analysis

The Statistical Package for the Social Sciences (SPSS) (version 25) was used to analyse the data. First of all, a descriptive statistical analysis was carried out in order to ascertain the state of the sample data. Subsequently, a cluster analysis was carried out to obtain information on the groups that were formed; these clusters were formed from the study variables of the PSDQ-S self-administered questionnaire [38]. In order to define the profiles and characteristics of the groups, the variables of the administered IPAQ questionnaire were used [40], as well as socio-demographic variables (age, gender, type of dwelling, and current residence). Using cluster analysis, we grouped those aspects of the sample in which they were heterogeneously grouped together. Firstly, outliers were calculated using the Mahalanobis distance, in which the distance (D) of each case to the centroid was calculated, eliminating those cases with an excessive distance, as recommended in the methodological literature [44,45], which requires strictness when classifying a case as an outlier (*p* < 0.001). Thus, in the present study, only 3 subjects were found and eliminated. After having detected and eliminated these outliers, a hierarchical cluster analysis was performed, using Ward’s agglomeration method, and as a measure of similarity, the squared Euclidean distance. Once this step was completed, the dendrogram was observed, which suggested the creation of 3 groups. Subsequently, a non-hierarchical analysis was carried out using K-means, where the three groups formed were indicated, in order to determine the correct grouping. Thus, in the results we found that the dendrogram performed in the non-hierarchical analysis, and also the 3 groups that were indicated to be performed in the non-hierarchical K-means analysis, with all the results obtained based on the application of the non-hierarchical K-means analysis.

## 3. Results

### 3.1. Cluster Analysis According to the PSDQ-S Instrument Dimensions

**Figure 1:**
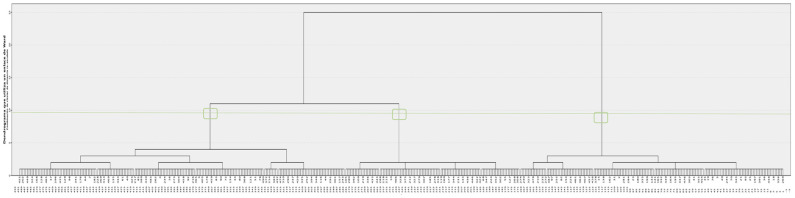
Cluster analysis dendrogram.

First of all, a hierarchical cluster analysis was carried out, which resulted in a dendrogram (Figure 1). The dendrogram describes the distribution of the data in each subcategory, according to the data being analysed. Cases that are similar will be grouped in each group and subgroup, thus creating the categories that the analysis considers relevant. In the present case, it can be seen that the hierarchical cluster analysis suggests creating three groups representing the totality of the cases, each divided by their similarity between each case, but distinct between each group.

#### Clusters Groups

After having defined the categories for the analysis, Table 1 shows the distribution of the cases in the three clusters created in the K-means analysis.

Once the distribution of the cases was carried out, the difference in the means of the clusters according to the variables or dimensions of the self-perception questionnaire (PSDQ-S) was identified in the analysis. As can be seen in Table 1, each group was assigned a name: Medium-Positive Physical Self-Concept (A±), Positive Physical Self-Concept (A+), and Negative Physical Self-Concept (A−). Before going on to detail the characteristics of each group, it is important to recognise that in the questionnaire not all the affirmative values, i.e., close to five points, are necessarily the best, since the variables of body fat, health, and in two items of global esteem, the scores that are closest to the value 1, will be the best valued. Therefore, the Medium-Positive Physical Self-Concept group (A±) is characterised by an average opinion in terms of the scores of the different items, with the best-valued variables being coordination, flexibility, esteem, and overall physical. In this group, the highest valued item is, “they feel confident when performing coordinated movements” (M = 3.83) and the lowest value is in the item that mentions daily sport practice, “I practice sport or physical activity almost every day” (M = 2.22).

The second group, defined as Positive Physical Self-Concept (A+), is characterised by having the best results both in the maximum 5-point values and in the values that are close to one, as these represent a better value despite being negative. This can be observed in the variable body fat, where the item, “I am overweight” with M = 1.88, would be the best valued, since it is not in agreement with that statement; the same happens in the variable health in the item, “I feel sick so often that I cannot do all the things I want to do” (M = 1.59), and in the variable global esteem in the item, “Nothing I do seems to work out well” (M = 1.77). It can also be identified that the variables of coordination (M = 3.56), overall physical (M = 3.17), and appearance (M = 3.13) have the highest overall positive mean values. Therefore, this group reflects that they feel physically well, with a high global esteem and, skilled in terms of flexibility, strength, and coordination, highlighting their positivism in comparison to the other groups.

On the other hand, regarding the third and last group called Negative Physical Self-Concept (A−), opinions with a negative tendency and values below three were reported in those items and dimensions whose positive affirmation should reach five points. Specifically, this is the case of the variable action, particularly in the item, “They practice sport or physical activity almost every day (M = 1.49), as well as in the variable appearance, “I am better looking than most of my friends” (M = 1.89). When it comes to dimensions such as body fat (M = 3.18) and health (M = 2.32), they have the highest overall mean values confirming their negativism compared to the other groups. Therefore, this group stands out for having a lot of body fat, not being resilient in terms of a physical test, not considering themselves flexible, neither good at sport, being not very quick to recover and illness prone, and not feeling happy with the appearance of their body, which reflects their overall low self-esteem, as the group with the most negative statements in terms of physical self-concept.

After analysing the mean values of each group in the different self-concept variables, ANOVA analyses were carried out to determine any significant differences between the groups in each of the elements of the analysis, using the Bonferroni post hoc test and Tamhane’s T2 test to verify in which groups these differences existed. Table 2 shows the differences found in most of the variables studied (*p* < 0.001), with no differences between groups A± and A−, in the flexibility variable, precisely in the items referring to, “My body is flexible” and “I think I would do well in a test that measures flexibility”. It was also possible to show that there were no significant differences in the health variable, specifically in the items, “I usually catch all kinds of illnesses” (influenza, colds, viruses, etc.); in the item, “I often get sick”; and in the item, “I have to go to the doctor more often for illnesses than other people of my age”. In this way, showing that there are significant differences in almost all the elements of the study, it is possible to demonstrate a correct conformation of each group. On the other hand, cross tables and a Chi-square analysis have been carried out to check if there is a significant relationship between the groups of clusters and the variables analysed with the three groups of clusters formed, with the aim of being able to analyse and identify in a more detailed and precise way, the most important characteristics of each of the groups. In order to obtain this more precise segmentation, the main aspects reflected by the analyses in each of the groups according to the questions of the IPAQ questionnaire are presented below.

### 3.2. Cluster Groups and Characterisation According to the Questions of the IPAQ Questionnaire

As for group A±, it can be observed in Table 3 that most individuals have not performed any intense activity in the last seven days, represented by 38.9%; however, 47.6% have performed some intense activity between one and three days a week, whereas 58.4% of individuals dedicated between fifteen and thirty minutes a day to this activity. In relation to the performance of moderate physical activity in the last seven days, 25.8% of individuals in this group indicate that they do not perform this activity, while 52.6% of individuals do perform moderate physical activity, dedicating between fifteen and thirty minutes a day to it, which is represented by 73.4% of individuals. In terms of daily walking in the last seven days, 55.1% of individuals in this group report that they mostly walk between one and three days, with 74.1% of individuals spending about fifteen to thirty minutes a day. Finally, 39.7% of individuals report sitting between one and three hours a day in the last seven working days, while 34% report sitting for more than six hours a day.

In the A+ group, 49.1% of individuals have performed intense activity between three and five days in the last week, of whom 75% of individuals spend between thirty minutes and one hour. On the other hand, 45.2% of individuals reported doing moderate physical activity between one and three days a week, with 36.3% of those who spent 30 min a day, while 16% of them did not do any such activity. As for walking, 18.9% of individuals walked every day of the week, followed by 44.4% who walked between one and three days. The majority (37.1%) spent 15 min walking, followed by 60.3% of individuals who spent between 30 min and one hour a day. Finally, it can be observed that in this group 35.5% of individuals have been sitting between one and three hours per week followed by 30.8% who have been sitting for more than six hours.

The last group, A−, indicates that 63.4% of individuals have not performed any intense physical activity, while 35.7% have done between one and three days in the last week, representing 67.4% of individuals who have spent between fifteen and thirty minutes a day. As for moderate activity, this group stands out for not doing this type of activity, representing 40.2% of individuals; however, 52.7% state that they have performed moderate activity between one and three days, with a majority (44.3%) of individuals dedicating fifteen minutes a day to this activity. On the other hand, 61.6% of individuals reported having walked between one and three days during the last week, with 44.3% of individuals dedicating thirty minutes a day to this activity. Likewise, it can be noted that the majority of this group (43.2%) were sitting for more than six hours a day in the last working week.

Finally, group A−, where 44.3% said they performed fifteen minutes of moderate activity during the day, followed by 27.1% who performed thirty minutes, leaving 12.9% and 11.4% who indicated that they exercised for one hour and forty-five minutes, respectively, on the day they performed moderate activity.

On the other hand, with regard to walking in the last seven days and with a minimum of 15 continuous minutes (Table 3), in group A±, the highest percentage of individuals (21.1%) said that they had performed this activity two days a week, followed by 17.7% who indicated having done it one day a week. Of the subjects, 16.3% walked three days a week, 14.4% the last seven days, and 10.5% did not do this activity in the last seven days. In relation to the minutes of walking, it was found that 39.2% of the individuals in group A± spent only fifteen minutes on the days they walked, followed by 34.9% who spent thirty minutes, leaving only 12.2% who spent between forty-five minutes and one hour walking (in both cases) on the days they carried out this activity. It can also be observed that in group A+ the highest percentage of individuals (18.9%), who walked at least fifteen minutes in a row, indicated that they had performed this activity seven days a week, followed by 17.8% who walked two days, while 13.6% walked only one day, and in a smaller proportion, 13% performed this activity three days a week. Regarding walking time in minutes, 37.1% of individuals reported walking for at least fifteen minutes. While 30.5% performed this activity for thirty minutes, and 15.9% and 13.9% spent forty-five and one hour a day, respectively.

With regard to group A−, it was found that the majority of individuals (23.2%) walked for at least fifteen minutes at a time on one day in the last week, followed by 19.6% who walked for two days at a time. A further 18.8% walked on three days, leaving 15.2% of the subjects who did not do this activity on the last seven days, leaving only 10.7% and 8% who walked on seven and four days, respectively. It was also observed that 44.3% said they had spent 30 min walking on the days they performed this activity, followed by 32% who said they had walked for fifteen minutes, 11.3% for forty-five minutes and, finally, 9.3% of the subjects for one hour.

Continuing with the description of the results reported by the IPAQ instrument (Table 3), it could be identified that, in group A± in the last seven days, 39.7% of the individuals remained seated between one and three hours a day, followed by 34% who claimed to have done so for more than six hours, in a lower proportion, 20.6% indicated having done so for between three and six hours, and only 5.7% did so for less than one hour a day. As for the A+ group, 35.5% said they had sat for between one and three hours a day, followed by 30.8% who sat for more than six hours a day, 27.8% who sat for between three and six hours, and only 5.9% who sat for less than one hour a day. Finally, with regard to group A−, 43.2% of individuals claimed to have sat for more than six hours a day, followed by 31.5% who sat for between one and three hours, 21.6% who sat for between three and six hours, and 3.6% who sat for less than one hour a day.

Finally, the comparison of means using the ANOVA test made it possible to identify in which variables and groups there were differences. The result of this statistical test reported significant differences (*p* > 0.001) between the variables: intense activities, minutes spent in intense activities, moderate activities, minutes spent in moderate activities, and walking for at least 15 min at a time. On the other hand, between the variables: daily minutes spent walking and minutes or hours spent sitting in the last seven days, no differences were found for a value of *p* < 0.05.

### 3.3. Cluster Groups and Characterisation According to Socio-Demographic Variables

With regard to the formation of clusters according to age range (Table 4), it can be seen that students under 30 years of age predominate in the three groups (group A± 91.4%, groups A+ 92.9%, and group A− 95.5%). It can then be seen that in the range between 31 and 45 years of age there is, in all three groups, a proportion of less than 7%.

In relation to the gender variable, it can be seen that in the three groups, the number of women predominates. In the A± group, women represent 65.6% compared to 34.4% of men, the A+ group is represented by 52.1% of women compared to 47.9% of men, being the most equal group in terms of gender. The A− group is represented by 77.75% of women, while the remaining 22.3% are men, being the group with the highest proportion of women.

With regard to the type of dwelling in which they live, in all three groups, the majority of individuals indicate that they live in a house. In particular, 74.2% of the A± group said that they lived at home, compared to 25.8% who said they lived in a flat. In the A+ group, 76.95% live at home and 23.1% live in a flat. Finally, regarding this variable, 79.5% of the A− group said they lived at home, compared to 20.5% who lived in a flat.

Continuing, and in relation to the geographical location of residence, the majority of individuals reported residing in the Valparaíso Region (group A± 84.2%, group A+ 77.55%, and group A− 83.9%), while 15.3% of individuals belonging to group A± reported residing in other regions, 21.3% of group 2 and 16.1% of group A−, in addition to a very low percentage of individuals who reported residing outside of Chile.

Finally, the results of the ANOVA test reported statistically significant differences only in the gender variable. In detail, the differences are between the A± group and the A+ group with a *p* = 0.003, and between the A+ and A− group with a *p*-value = 0.001.

## 4. Discussion

In the university environment, it is of great importance to know the groups that can be formed among students, and thus to be able to direct more effectively the strategies for the promotion of physical-sports programmes, and to place emphasis on promoting the participation of young people in physical activity [46]. This is justified, considering the post-pandemic scenario and the benefits it can provide to university students, as it influences relevant predictors of their physical and mental health [47].

In this sense, the present study, after the hierarchical cluster analysis of the studied sample, was able to establish three different group profiles of university students, which were denominated: Medium-Positive Physical Self-Concept (A±), Positive Physical Self-Concept (A+), and Negative Physical Self-Concept (A−). These three groups showed significant differences in all the self-concept variables studied (physical activity, appearance, body fat, coordination, endurance, flexibility, health, sport, strength, global physical, and global esteem), only with no differences in some of the flexibility items between the A± group and the A− group and in some of the health items between the A± and A− groups. Therefore, these student profiles should be considered in order to be able to orientate different strategies for promoting activities, which are adapted to each of them, so that those students who are not very active can carry out different activities and, consequently, have a better self-concept. The A± group is characterised by showing an average opinion regarding all the self-concept variables, standing out with higher scores in the variables of coordination, flexibility, global esteem, and global physical; that is to say, they contribute averages between 2 and 4 points on the Likert scale, which reflects that they have an average self-concept. Group A+ is characterised by the highest scores on all variables; this group has an elevated self-concept, and stands out from the other groups. Group A−, is characterised by having opinions with a negative tendency and values below three points; this group stands out for having a low self-concept in comparison to the other groups similar to the results obtained in the work of Hossain et al. [47].

The composition of groups according to the IPAQ questionnaire shows that the three groups are clearly differentiated with regard to the level of physical activity. It was possible to identify that in group A± there are individuals who carry out physical activity, in this group average values of activity were obtained, as there were individuals who carried out some activity and others who did not, and average values were presented in this aspect. As for the A+ group, they are those who carry out the most physical activity (intense, moderate, and low), and also stand out as those who dedicate the most minutes per day to intense, moderate, and walking activities, along the same lines as the findings of the works of Dieppa et al. [48] and Contreras et al. [49]. Notably for this group, which also reported higher levels of physical self-concept than a pre-pandemic study, a positive correlation was found between this aspect and higher levels of physical activity [28] and along the same lines as concluded by Guillamón, García, and Carrillo [50]. Finally, group A−, stands out for being the least active (intense, moderate, and low), this group is the one in which the highest number of individuals did not perform any physical activity in the last 7 days, as well as the group that walked the least minutes and days per week, which may be associated with the scenario of the COVID-19 pandemic during the year 2020 and the social isolation and/or quarantine that had a significant impact on the levels of physical activity and exercise [51,52].

Among the three groups, there were no significant differences in terms of low-intensity physical activities (walking and sitting); although, there were such differences between physical activity and inactivity, where in all three groups, there was an equality between subjects who spent more than 6 h a day sitting and did not walk more than 30 min a day.

As a general conclusion of the self-concept questionnaire and the “IPAQ”, it can be said that the Positive-Physical Self-Concept group is the most physically active, being the most equal group between men and women, and the group with the highest self-concept. While the Physical-Medium Self-Concept and Physical-Negative Self-Concept groups are the least physically active, both groups are predominantly female and reflect a lower self-concept. In line with the findings of a study carried out during the pandemic [15], and showing a similar trend to that observed by Navas and Soriano [53] in their work prior to the pandemic. This could be attributed to social factors, such as stereotypes and prejudices that exist in the community [54], which generally affect women more than men.

On the other hand, in the composition of the groups, and according to the socio-demographic variables, it could be seen that most of the students in each of the groups are under 30 years of age; likewise, the female gender predominated in all of them, being the A− and A+ groups where women represent a higher percentage of the sample.

## 5. Conclusions

In the ANOVA analysis, it was concluded that there were statistically significant differences between the three groups in all the variables of the physical self-concept (*p* < 0.001), with only a few items remaining without such differences. Likewise, in the analysis carried out on the IPAQ items, there were statistically significant differences in several of the items (intense activities, minutes dedicated to intense activities, moderate activities, minutes dedicated to moderate activities, and walking for at least 15 min at a time). Finally, with regard to the sociodemographic variables, differences were found only in the sex variable.

## 6. Limitations

The results of this research cannot be generalizable, as a non-probabilistic convenience sample was considered because of the students to whom access was available. The measurements taken to determine participants’ physical activity levels using the IPAQ may have limitations of recall bias, as it requires differential reporting of time spent on different types of activities in the last 7 days, as well as it being a self-reporting technique.

## Figures and Tables

**Table 1:** Established clusters.

Group	%	*n*
1. Medium-Positive Physical Self-Concept (A±)	36%	176
2. Positive Physical Self-Concept (A+)	40%	196
3. Negative Physical Self-Concept (A−)	24%	117

**Table 2:** Mean physical self-concept ratings.

Item	A±		A+		A−		*p*	*p*	*p*
	M	SD	M	SD	M	SD	A± vs. A+	A± vs. A−	A+ vs. A−
I often do demanding exercise or physical activities	2.60	1.19	3.82	1.08	1.63	0.85	***	***	***
I do a lot of physical activity (e.g., jogging, dancing, cycling, aerobics, swimming, gymnastics) at least three times a week	2.56	1.33	3.80	1.35	1.64	0.90	***	***	***
I do a lot of sport, dancing, gymnastics and other physical activities	2.56	1.13	3.96	1.13	1.59	0.73	***	***	***
I practice sports or physical activity almost every day.	2.22	1.33	3.72	1.32	1.49	0.81	***	***	***
Appearance									
I have a nice face	3.68	0.90	4.11	8.31	2.46	1.03	***	***	***
I am more handsome than most of my friends	2.83	0.97	3.46	1.02	1.89	0.94	***	***	***
I am quite graceful	3.50	0.93	4.07	0.90	2.25	1.03	***	***	***
Body Fat									
My abdominal girth is very large	2.60	1.14	2.02	9.85	3.19	1.35	***	***	***
I feel like I have a lot of body fat	3.09	1.22	2.46	1.14	4.04	1.14	***	***	***
I am overweight	2.61	1.51	1.88	1.32	3.46	1.63	***	***	***
Coordination									
I feel confident when I am making coordinated movements	3.83	0.95	4.48	0.73	2.96	1.19	***	***	***
It is easy for me to control my body movements	3.72	0.94	4.28	0.93	3.19	0.98	***	***	***
I am good at movements that require coordination	3.43	1.08	4.05	0.89	2.62	1.13	***	***	***
I can make slow, smooth and fluid movements in most physical activities.	3.59	0.91	4.28	0.75	3.15	1.04	***	***	***
I think my body adapts well to coordinated movements.	3.49	0.98	4.24	0.81	2.75	1.05	***	***	***
Resistance									
I can run for a long time without stopping	2.69	1.14	3.81	1.11	1.90	1.01	***	***	***
I can be physically active without getting tired	2.75	0.94	4.05	0.85	1.95	0.94	***	***	***
I am good at endurance events e.g., running, aerobics, swimming, cross country, skiing, etc.	2.44	1.06	3.56	1.06	1.63	0.87	***	***	***
Flexibility									
I am able to bend, move and flex my body	3.37	1.08	3.91	1.04	2.89	1.26	***	***	***
My body is flexible	3.04	1.12	3.68	1.10	2.74	1.24	***	-	***
I think it would do well in a flexibility test.	2.82	1.08	3.61	1.04	2.52	0.90	***	-	***
Health									
I usually catch all illnesses (influenza, colds, viruses, etc.)	1.68	0.92	1.53	0.83	2.19	1.27	-	***	***
I feel sick so often that I can’t do all the things I want to do	1.59	0.90	1.41	0.78	2.27	1.29	***	***	***
I frequently get sick	1.62	0.92	1.36	0.70	2.28	1.25	-	***	***
When I get sick, it takes me a long time to feel well	1.94	0.97	1.68	0.83	2.42	1.18	**	***	***
I have to go to the doctor more often for illnesses than other people my age	1.67	1.07	1.50	0.90	2.32	1.39	-	***	***
Sport									
I am good at most sports	2.54	1.03	3.69	0.94	1.75	0.84	***	***	***
I have good sports skills	3.07	0.99	4.28	0.76	2.07	0.87	***	***	***
I play sports well	2.85	1.03	3.99	0.93	1.87	0.95	***	***	***
Strength									
Physically I am a strong person	3.07	1.00	3.89	0.99	2.09	0.97	***	***	***
I have a lot of power in my body	3.37	0.92	4.25	0.76	2.21	0.99	***	***	***
It would do well in a strength test	2.72	1.09	3.63	1.14	1.68	0.85	***	***	***
Global Physical									
I feel happy with myself physically	3.28	1.00	4.06	0.98	1.95	0.82	***	***	***
Physically I feel good about myself	3.21	0.99	4.06	0.94	1.79	0.82	***	***	***
I feel good about who I am physically	3.27	0.93	4.18	0.86	1.88	0.88	***	***	***
Global Estimate									
Most of all, most of the things I do work out well	3.29	0.81	4.07	0.71	2.69	0.86	***	***	***
In general, I am not very good at anything	1.82	0.95	1.40	0.80	3.13	1.22	***	***	***
Most of the things I do, I do well	3.43	0.76	4.07	0.77	2.51	0.91	***	***	***
I have a lot to be proud of	3.79	0.93	4.47	0.76	2.63	1.08	***	***	***
Nothing I do seems to go right	1.77	0.88	1.33	0.61	2.82	1.11	***	***	***

Note: *** *p* < 0.001; ** *p* < 0.05; SD = Standard Deviation; A± = Medium-positive Physical Self-Concept; A+ = Positive Physical Self-Concept; A− = Negative Physical Self-Concept; M = mean; *p* = One-factor ANOVA.

**Table 3:** Groups according to questions in the IPAQ questionnaire.

Intensity	Frequency	*n*	A±	*n*	A+	*n*	A−	*p* A± vs. A+	*p* A± vs. A−	*p* A+ vs. A−
INTENSIVE physical activities in the last 7 days	NO physical activity	81	38.9%	25	14.8%	71	63.4%	***	***	***
	1 day	42	20.2%	14	8.3%	15	13.4%			
	2 days	20	9.6%	24	14.2%	14	12.5%			
	3 days	37	17.8%	49	29.0%	11	9.8%			
	4 days	0	-	0	0.0%	0	-			
	5 days	23	11.1%	34	20.1%	1	0.9%			
	6 days	4	1.9%	16	9.5%	0	-			
	7 days	1	0.5%	7	4.1%	0	-			
Daily minutes spent in INTENSIVE activities	15 min	35	25.5%	21	13.8%	16	34.8%	**		***
	20 min	2	1.5%	7	4.6%	1	2.2%			
	30 min	43	31.4%	30	19.7%	14	30.4%			
	45 min	24	17.5%	32	21.1%	8	17.4%			
	1 h	27	19.7%	52	34.2%	7	15.2%			
	1 h 30 min	4	2.9%	7	4.6%	0	-			
	2 h or more	2	1.5%	3	2.0%	0	-			
MODERATE physical activities in the last 7 days	NO physical activity	54	25.8%	27	16.0%	45	40.2%	***	**	***
	1 day	41	19.6%	23	13.6%	20	17.9%			
	2 days	36	17.2%	25	14.8%	24	21.4%			
	3 days	33	15.8%	29	17.2%	15	13.4%			
	4 days	17	8.1%	17	10.1%	2	1.8%			
	5 days	18	8.6%	16	9.5%	6	5.4%			
	6 days	5	2.4%	12	7.1%	0	-			
	7 days	5	2.4%	20	11.8%	0	-			
Daily minutes spent in MODERATE activities	15 min	51	32.3%	29	19.9%	31	44.3%	**		***
	20 min	0	-	3	2.1%	3	4.3%			
	30 min	65	41.1%	53	36.3%	19	27.1%			
	45 min	24	15.2%	27	18.5%	8	11.4%			
	1 h	17	10.8%	28	19.2%	9	12.9%			
	1 h 30 min	0	-	4	2.7%	0	-			
	2 h or more	1	0.6%	2	1.4%	0	-			
Walking in the last 7 days	NO physical activity	22	10.5%	20	11.8%	17	15.2%			**
	1 day	37	17.7%	23	13.6%	26	23.2%			
	2 days	44	21.1%	30	17.8%	22	19.6%			
	3 days	34	16.3%	22	13.0%	21	18.8%			
	4 days	23	11.0%	19	11.2%	9	8.0%			
	5 days	16	7.7%	13	7.7%	5	4.5%			
	6 days	3	1.4%	10	5.9%	0	-			
	7 days	30	14.4%	32	18.9%	12	10.7%			
Minutes per day spent walking	15 min	74	39.2%	56	37.1%	31	32.0%			
	20 min	1	0.5%	0	0.0%	1	1.0%			
	30 min	66	34.9%	46	30.5%	43	44.3%			
	45 min	23	12.2%	24	15.9%	11	11.3%			
	1 h	23	12.2%	21	13.9%	9	9.3%			
	1 h 30 min	2	1.1%	2	1.3%	0	0.0%			
	2 h or more	0	0.0%	2	1.3%	2	2.1%			
Minutes or hours per day spent sitting in the last 7 working days	<1 h	12	5.7%	10	5.9%	4	3.6%			
	Between 1 and 3 h	83	39.7%	60	35.5%	35	31.5%			
	Between 3 and 6 h	43	20.6%	47	27.8%	24	21.6%			
	>6 h	71	34.0%	52	30.8%	48	43.2%			

Note: A± = Average-positive Physical Self-Concept; A+ = Positive Physical Self-Concept; A− = Negative Physical Self-Concept; *** *p* < 0.001; ** *p* < 0.05; *n* = number of subjects; *p* = One-factor ANOVA.

**Table 4:** Groups according to socio-demographic variables.

Variable	Response	*n*	A±	*n*	A+	*n*	A−	*p* A± vs A+	*p* A+ vs A−
Age by groups	Up to 30 years	191	91.4%	157	92.9%	107	95.5%		
	Between 31 and 45 years	14	6.7%	8	4.7%	4	3.6%		
	over 46 years	4	1.9%	4	2.4%	1	0.9%		
Gender	Male	72	34.4%	81	47.9%	25	22.3%	**	***
	Female	137	65.6%	88	52.1%	87	77.7%	**	***
Type of residence in which you live	House	155	74.2%	130	76.9%	89	79.5%		
	Flat	54	25.8%	39	23.1%	23	20.5%		
Current residence	Valparaíso	176	84.2%	131	77.5%	94	83.9%		
	Other regions	32	15.3%	36	21.3%	18	16.1%		
	out of Chile	1	0.5%	2	1.2%	0	-		

Note: A± = Average-positive Physical Self-Concept; A+ = Positive Physical Self-Concept; A− = Negative Physical Self-Concept; *** *p* < 0.001; ** *p* < 0.05; *n* = Number of subjects.

## Data Availability

Not applicable.
